# Jaw mobility changes in patients with upper aerodigestive tract cancer undergoing radiation therapy

**DOI:** 10.4317/medoral.20477

**Published:** 2015-10-09

**Authors:** Karoline Bragante, Patrícia Wienandts, Carolina Mozzini, Rosélie Pinto, Neiro da Motta, Geraldo Jotz

**Affiliations:** 1 PT, MSc. Investigator, Graduate Program in Health Sciences, Universidade Federal de Ciências da Saúde de Porto Alegre (UFCSPA), Porto Alegre, Rio Grande do Sul, Brazil. Department of Radiation Oncology, Santa Rita Hospital, Irmandade Santa Casa de Misericórdia de Porto Alegre (ISCMPA), Porto Alegre, Rio Grande do Sul, Brazil. Department of Health Science and Rehabilitation, UFCSPA, Porto Alegre, Rio Grande do Sul, Brazil; 2 DDS, MSc. Investigator, Department of Special Dental Care, ISCMPA, Porto Alegre, Rio Grande do Sul, Brazil; 3 PT, PhD. Investigator, Department of Health Science and Rehabilitation, UFCSPA, Porto Alegre, Rio Grande do Sul, Brazil; 4 RN. Head Nurse, Department of Radiation Oncology, Santa Rita Hospital, ISCMPA, Porto Alegre, Rio Grande do Sul, Brazil; 5 MD, PhD. Chief, Department of Radiation Oncology, Santa Rita Hospital, ISCMPA, Porto Alegre, Rio Grande do Sul, Brazil; 6 MD, PhD. Associate Professor, Graduate Program in Health Sciences, UFCSPA, Porto Alegre, Rio Grande do Sul, Brazil

## Abstract

**Background:**

Radiation therapy is a therapeutic modality widely used for treatment of upper aerodigestive tract (UADT) neoplasms. However, its action is not restricted to tumor cells, and it may cause a variety of adverse reactions, including reduced jaw mobility.

**Material and Methods:**

A prospective cohort study was conducted to assess changes in jaw mobility in patients with UADT cancer undergoing radiation therapy.

**Results:**

Fifty-six patients completed the study. The results showed a significant reduction in mouth opening (*p*<0.001), right lateral excursion (*p*=0.038) and left lateral excursion (*p*=0.035) of the jaw, a significant increase in the presence (*p*<0.001) and severity of oral mucositis (*p*<0.001), and a significant decrease in performance status (*p*<0.001) after radiation therapy. Thirty-six patients (64.3%) exhibited reduction in mouth opening after treatment. The variables significantly associated with mouth opening reduction on bivariate analysis were: modification of diet (*p*=0.037), radiation field (*p*=0.024), presence of mucositis (*p*=0.003), and reduction in performance status (*p*=0.007). After adjustment by the multivariate model, the only variables that remained significantly associated with reduction in mouth opening were presence of mucositis (*p*=0.018) and reduction in performance status (*p*=0.47).

**Conclusions:**

These findings indicate that patients with upper aerodigestive tract cancer experience reduced jaw mobility after radiation therapy, which is strongly correlated with mucositis and reduced functional ability.

**Key words:**Head and neck neoplasms, vertical dimension, radiation therapy, mucositis, temporomandibular joint, joint range of motion, trismus.

## Introduction

Over 1 million cases of upper aerodigestive tract (UADT) cancer are diagnosed worldwide each year, a number that is expected to increase (1). Oral cavity cancer is the seventh most common type of cancer in the Brazilian population (2), and the male population of Brazil has the third-highest risk of oral cancer in the world, after those of France and India (2).

According to the literature, normal jaw mobility ranges from 40 to 70 mm for mouth opening and from 8 to 12 mm for lateral excursion (3,4). However, these values can vary with factors such as gender, age, body weight, height, dental conditions, and signs and symptoms of temporomandibular disorders (4). UADT cancer-related reduced jaw mobility, or trismus, may occur due to tumor infiltration into the muscles of mastication or into the temporomandibular joint (TMJ). Other causes include mechanical obstruction of the coronoid process of the mandible by the tumor, surgical approaches to tumors involving the muscles of mastication or TMJ, adhesions and scar-related fibrosis, and radiation-induced fibrosis (5).

The incidence of mandibular hypomobility among UADT cancer patients treated with radiation therapy ranges from 6% to 85% (6-9). This broad range seems to be related with biases in retrospective collections, lack of uniform criteria to measure this complication, different anatomy sites and tumor sizes, and application of different forms of treatment and irradiation modes (5,7,10). Reduction in jaw mobility negatively impacts patient quality of life, as it changes facial appearance, hinders food intake and use of dental prostheses, compromises oral hygiene and speech, and may induce anxiety and depression (7-9).

In view of the lack of reliable data on the incidence of mandibular hypomobility among irradiated UADT cancer patients, the primary objective of this study was to assess jaw mobility in a cohort of patients with UADT cancer undergoing radiation therapy at a charitable hospital in Porto Alegre, state of Rio Grande do Sul, Brazil. The secondary objective was to ascertain whether changes in jaw mobility are associated with selected demographic and clinical variables.

## Material and Methods

This prospective cohort consecutively assessed all patients with UADT cancer - defined for the purposes of this study as cancer of the oral cavity, oropharynx, nasopharynx, hypo pharynx, or larynx - who attended the Department of Radiation Oncology of a charitable hospital in Porto Alegre, Brazil, between January 1st and June 30th, 2013.

Subjects included persons over 18 years of age, of either gender, who had received an anatomopathological diagnosis of UADT cancer and were undergoing radiation therapy (RT) exclusively or in association with chemotherapy and/or surgery. Patient selection was limited to those whose TMJ and/or muscles of mastication were in the radiation field and who were receiving RT with curative intent. The following exclusion criteria were applied: Surgical interventions that involved removal of the jaw or any of the muscles of mastication, facial palsy, trigeminal neuralgia, previous irradiation of the head and neck region, current physical therapy or speech therapy for jaw mobility, baseline mouth opening (MO) less than 10 mm, score below 50 on the Karnofsky Performance Status (KPS) scale (11), and refusal to take part in the study.

Sample size calculation was based on a previous study by Grandi *et al*. (12). We estimated that at least 43 patients would be required to detect an effect size of at least 0.6 standard deviations between assessments, at the 5% significance level, with 90% power.

- Data Collection

Eligible patients were identified before the start of RT, during the weekly multidisciplinary team meeting of the Department of Radiation Oncology. On the day of the patient’s treatment simulation, the study was explained and patients were invited to participate. After each patient provided written informed consent, data collection was begun.

All patients underwent individual history-taking and physical examination, always performed by the same examiner, who was qualified and experienced with the research protocols at hand. Data on the following variables were collected: age, gender, skin color, occupation, family history of cancer, tobacco and alcohol intake, and consistency of diet (solid, semisolid, liquid, or enteral nutrition).

Occupational exposure to carcinogenic substances was assessed according to the description of the Union for International Cancer Control (UICC) (13). When the patient had a positive family history of cancer, the disease was classified into UADT cancer, tobacco-related cancer (lung, esophageal, liver, stomach, spleen, kidney, bladder, cervix, and myeloid leukemia), or other cancer types (14).

The clinical data collected were: tumor site, histologic type, concomitant chemotherapy, surgery, modality of RT, radiation field and dosage and tumor staging. These data were collected from patient records.

Physical examination was performed prior to the start of RT (pre-treatment) and immediately after the last RT session (post-treatment), and assessed maximum mouth opening (MMO), right lateral excursion (RLE), left lateral excursion (LLE), and protrusion (PR) of the jaw, oral mucositis, and functional ability.

Jaw MMO, RLE, LLE, and PR were measured with a Digimess 100.179N digital caliper (resolution 0.01 mm/.0005”, with a certificate of calibration provided by the manufacturer), following the guidelines and specifications for clinical examination defined by the Research Diagnostic Criteria for Temporomandibular Disorders (RDC/TMD) (15). The adaptations proposed by Goldstein *et al*. (6) for the assessment of partially and completely edentulous patients were adopted. In patients with an edentulous mandible who did not wear dentures, the distance from the incisal edge of the vertical most maxillary central incisor to the opposing alveolar ridge in the mandible was measured. In patients with an edentulous maxilla who did not wear dentures, the distance from the incisal edge of the vertical most mandibular central incisor to the opposing alveolar ridge was measured. In completely edentulous patients, MMO was measured from the mandibular alveolar ridge to the maxillary alveolar ridge at the mid line corresponding to the nasopalatine foramen.

Oral mucositis was graded according to the oral toxicity criteria of the World Health Organization (WHO) (16), while functional ability was assessed using the KPS scale (11).

All patients were treated with external photon beam RT. All received a daily dose of 2 Gy per fraction, 5 days a week, over a 5-to-7-week course of therapy. The total RT dose ranged from 50 to 70 Gy. Thirty-one (55.4%) patients received concomitant chemotherapy (50 mg cisplatin weekly). Twenty-two (39.3%) patients underwent surgical resection of the primary tumor. The treatment regimens were defined according to the tumor site and size and the disease stage.

The present study complied with the ethical standards of the Declaration of Helsinki and with Brazilian National Health Council Resolution no. 196/96, and was approved by the Research Ethics Committee of Irmandade Santa Casa de Misericórdia de Porto Alegre under protocol 031.12.

- Statistical Analysis

Quantitative variables were described as means and standard deviations or, if asymmetrically distributed, as medians and interquartile ranges. Categorical variables were described as absolute and relative frequencies.

The Shapiro-Wilk test was used to assess the distribution of variables, while Student’s T-test was used to compare means among groups.

To assess the association among quantitative variables, Pearson’s (r) or Spearman’s (rs) linear correlation coefficients were used for symmetrically and asymmetrically distributed data respectively.

Student’s T-test for paired samples was used to compare pre- and post-RT parameters. In case of distribution asymmetry, the Wilcoxon test was used instead. McNemar’s test was used to compare the proportions.

To control for confounding factors, a multivariate multiple linear regression model was applied. The criterion for inclusion of variables in the multivariate regression model was *p*<0.20 at bivariate analysis.

The significance level was set at 5% (*p*<0.05). All analyses were carried out in the SPSS 21.0 software environment.

## Results

Data were collected from 58 patients, two of whom were lost to follow-up (one deceased, one discontinued treatment as advised by physician). The demographic and clinical profiles of the 56 patients that completed the study are shown in  Table 1 and  Table 2 respectively.

**Table 1:**
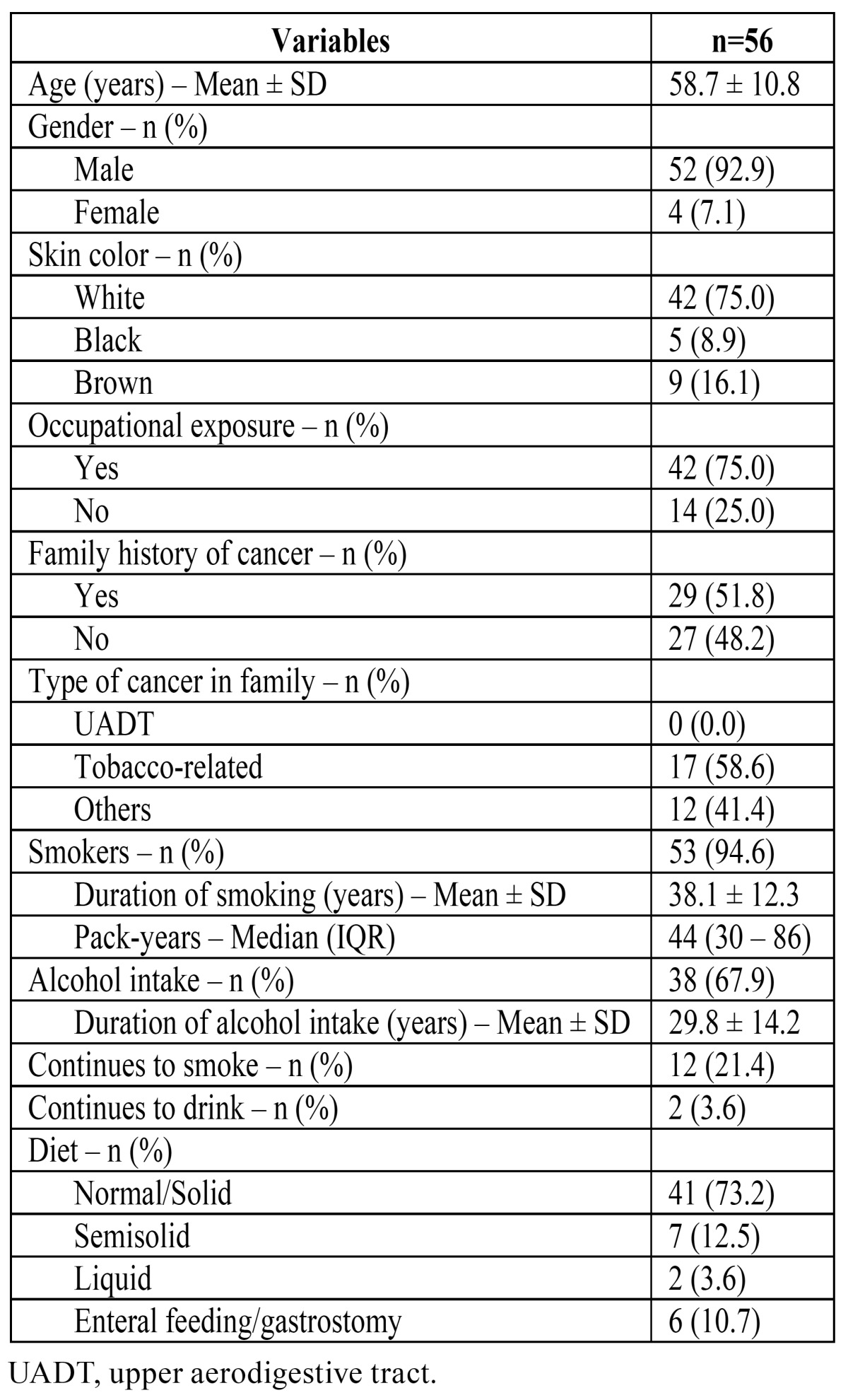
Sample profile.

**Table 2:**
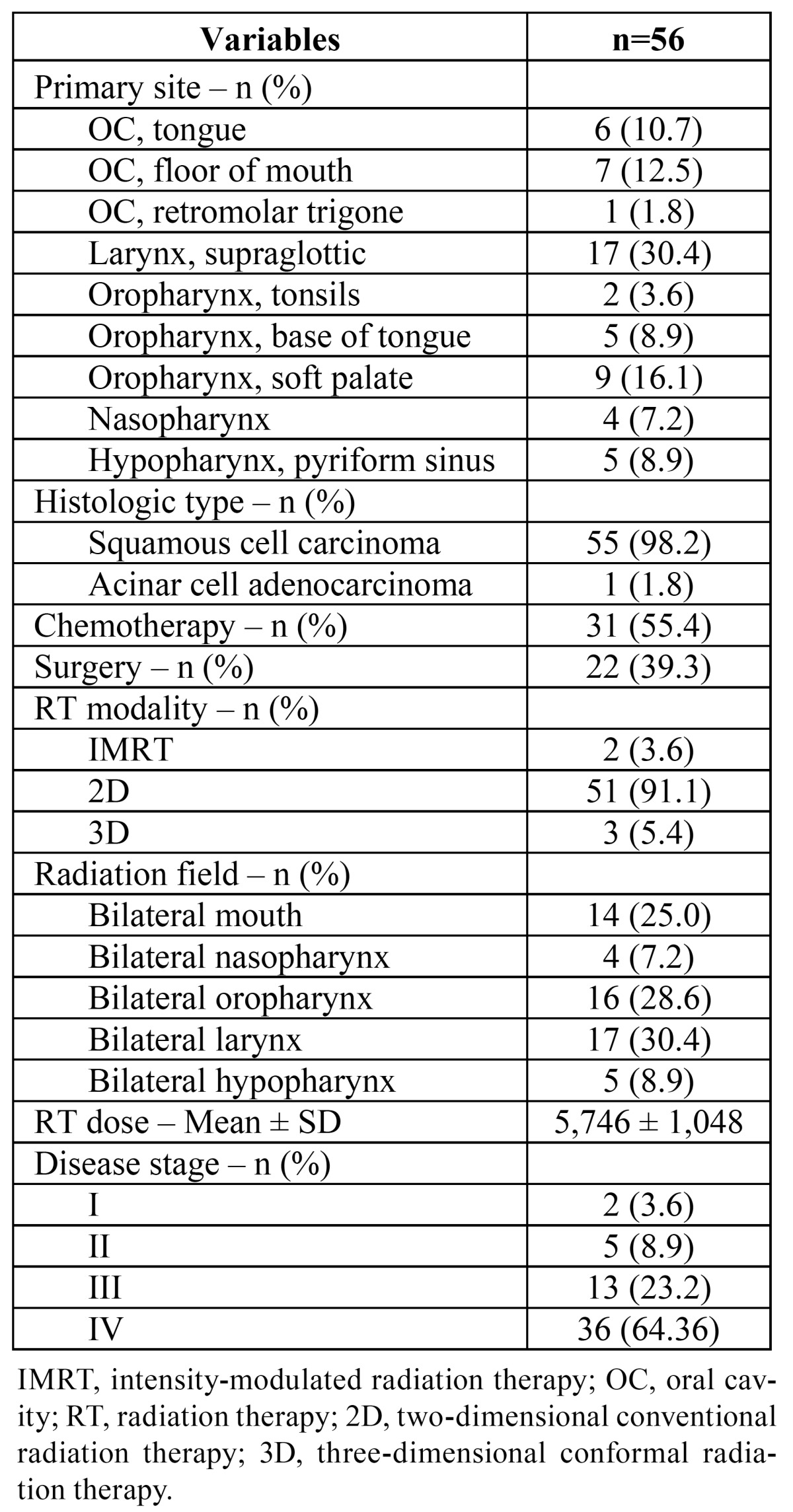
Clinical characteristics.

Comparison of the pre- and post-treatment physical examination variables of the 56 patients who completed the study showed a statistically significant reduction in MO, RLE, and LLE, a statistically significant increase in the presence and severity of mucositis, and a statistically significant decline in functional ability ( Table 3). After RT, MO decreased in 36 (64.3%) patients, increased in 12 (21.4%), and remained stable in eight (14.3%).

**Table 3:**
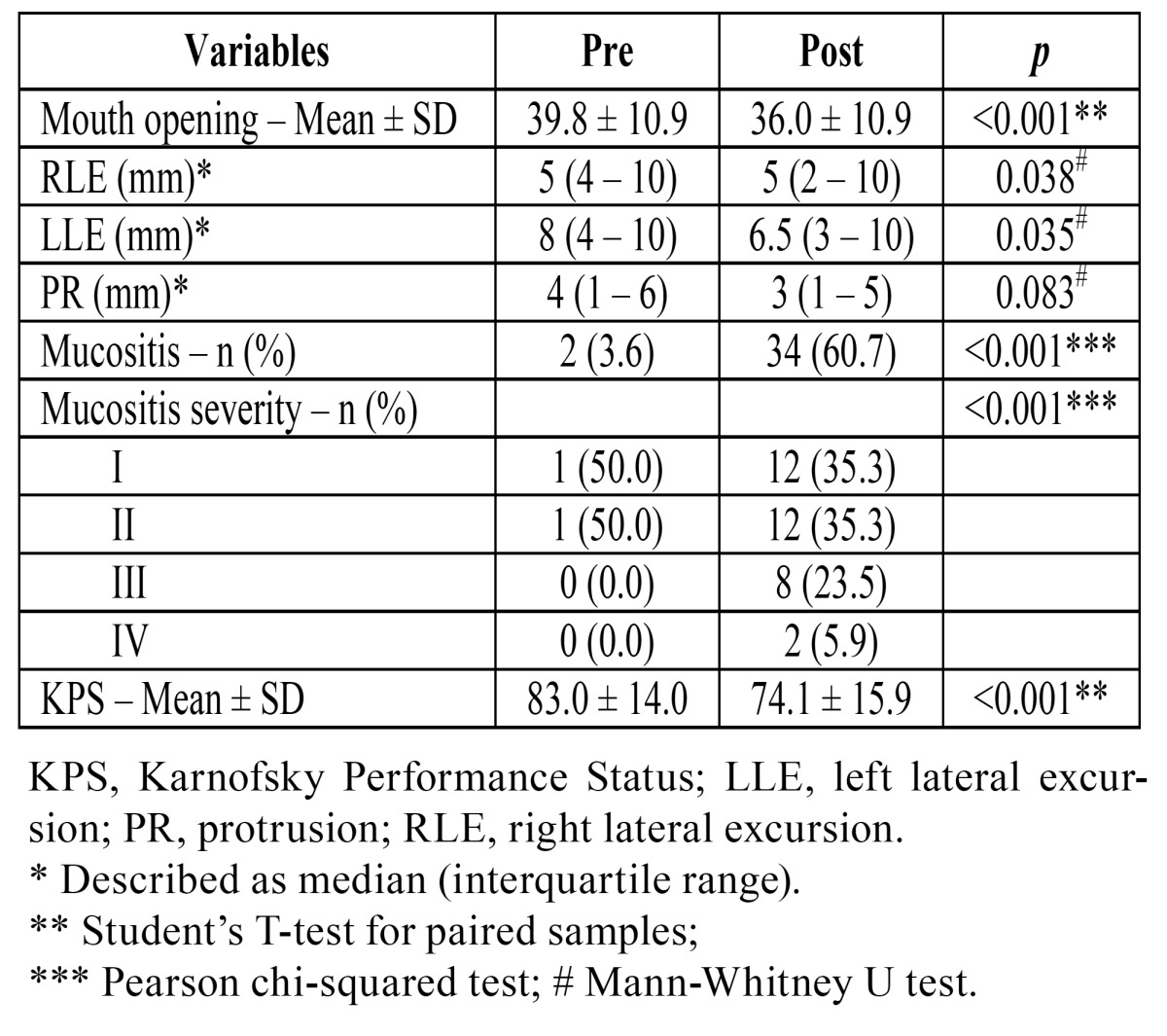
Pre- and post-radiation therapy comparison.

Bivariate analysis of clinical and demographic variables and physical examination findings showed that the following variables were significantly associated with change in MO: change in diet consistency (solid, -1.35±5.17 vs. modified, -5.17±7.00; *p*=0.037); radiation field (oral cavity/oropharynx, -5.64±6.42 vs. nasopharynx/hypo pharynx/larynx, -1.68±6.27; *p*=0.024); presence of mucositis (present, -5.86±6.58 vs. absent, -0.62±5.34; *p*=0.003); and reduction in KPS score (r=0.438, *p*=0.007).

Patients who had mucositis had a significantly higher rate of modified diet as compared with those who had no mucositis (82.4% vs. 20.6%, *p*=0.003).

After adjustment using the multivariate model ( Table 4), the only variables that remained significantly associated with MO reduction were presence of mucositis at post-RT assessment and reduction in KPS score. Patients with mucositis had an average reduction of 4.19 mm in MO after RT (b=-4.19; 95%CI -7.62 to -0.8). Patients whose KPS score decreased by 1 point also had a mean reduction of 0.12 mm in MO (b=0.12; 95%CI 0.02 to 0.24). On analysis of the standardized regression coefficient (β), the variable most strongly associated with reduction in MO was presence of mucositis.

**Table 4:**
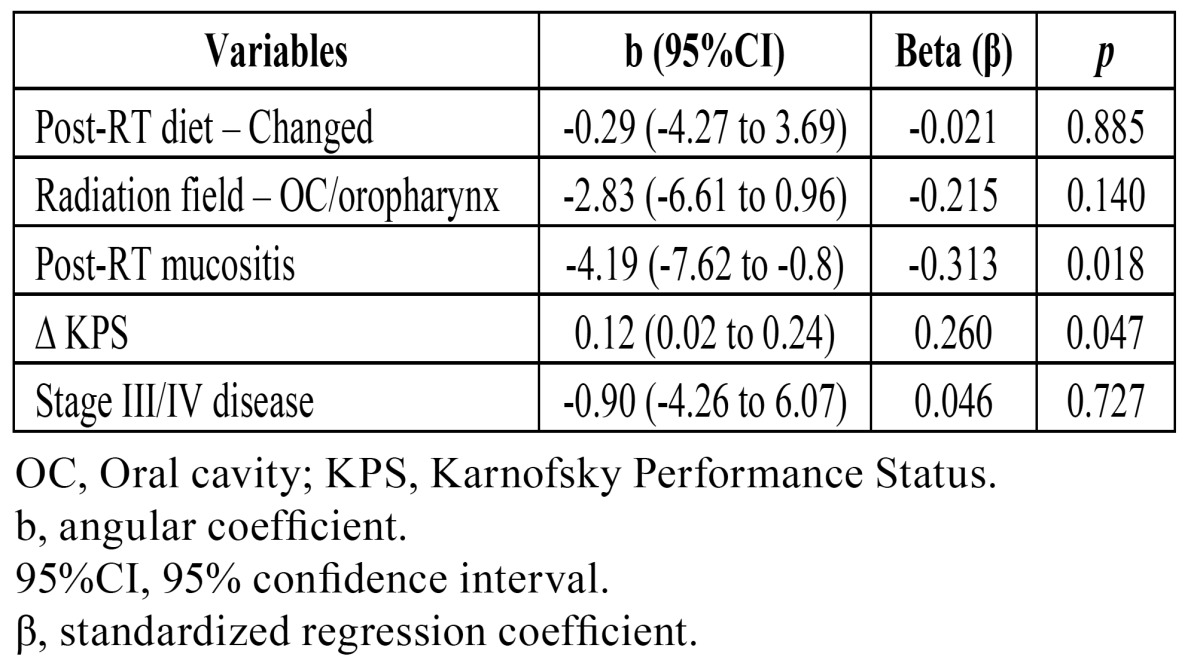
Multivariate linear regression analysis of factors associated with a reduction in mouth opening.

## Discussion

Regarding sociodemographic and clinical variables, the results of the present study are consistent with the existing literature, which reports a higher incidence of UADT cancer among men, with a predominance of individuals in the 6th and 7th decades of life and white males (2,8,12,17). Habits and social factors reported in other studies, such as alcohol and tobacco intake, tobacco-related cancers in the family, occupational exposure to carcinogenic substances, and low socioeconomic level (16,18,19), were also found in the present investigation. All patients who underwent two-dimensional conventional radiation therapy (2D-RT) (91%) were treated through the public healthcare system, in contrast with the 9% who used private health insurance or paid out of pocket and had access to three-dimensional conformal radiation therapy (3D-RT) and intensity-modulated radiation therapy (IMRT). The most prevalent histologic tumor type in this sample was squamous cell carcinoma, which is in accordance with the Brazilian cancer registry (2). Most patients were at an advanced stage at the time of clinical diagnosis, as also reported in other studies (8,17).

The patients in this study experienced a high incidence (64.3%) of reduced jaw mobility after RT, which corroborates the reports of previous studies with the same prospective design (6,20). A high incidence of mandibular hypo mobility was also found in two retrospective studies (17,21) and one cross-sectional study (8). However, none of those studies assessed mucositis (which was the variable most strongly associated with hypo mobility in this study) or the occurrence of mucositis in relation to the reduction in MO.

By causing pain, mucositis limits the functions of the stomatognathic system, which also forces patients to change the consistency of their diet and, often, to adopt exclusive enteral feeding (22). Patients thus move their jaws less and less, which results in connective tissue contractures and deterioration of the muscles of mastication (23). Muscles start to show signs of atrophy after as little as 3 days of restricted range of motion (5). It is important to remember that immobile joints also undergo rapid degenerative changes, including thickening of the synovial fluid and thinning of the cartilage (5). Thus, patients fed via the nasoenteral route may not notice the slow and progressive onset of trismus until they try to resume solid food intake, since trismus is a complication often neglected by staff involved in patient care and follow-up (5).

The incidence of mucositis is around 80% among irradiated UADT cancer patients. Both the incidence and severity of mucositis may increase if chemotherapy is administered concomitantly with RT (24), which will decrease jaw mobility. Nevertheless, concomitant chemotherapy was not associated with a reduction in MO in the present study, a finding that corroborates previous reports (21,23).

Mucositis requires early intervention, as the mucous membranes are the first structures to be harmed by radiation and, despite 90-95% of patients recovering from mucositis within 4 weeks of treatment completion (25), the effect of mandibular hypo mobility caused by mucositis will have a negative impact on the muscles of mastication and TMJ. This impact is difficult to reverse completely at later stages, especially if fibrosis develops in the affected muscles. As noted above, immobile joints rapidly undergo degenerative changes that can make remobilization difficult (5).

Radiation-induced fibrosis of the muscles of mastication as a cause of late post-RT mandibular hypo mobility usually occurs 9 weeks after the end of the treatment and progresses over a period of 6 to 9 months (6,9). It has been detected as late as 4 years after the end of treatment (26), which is explained by the fact that muscles are slow-responding tissues that exhibit signs of damage long after RT (27). However, as the present study demonstrated, jaw mobility may also decrease during RT, particularly if mucositis occurs.

Studies assessing reduction in jaw mobility during RT have found that range of MO had not recovered at 6 months after treatment (9,20), while one study showed that MO remains reduced over several years (26). Protocols for prevention of mucositis and radiation-induced trismus must be tested to ascertain whether prophylactic interventions are able to preserve jaw mobility during RT or, at least, mitigate loss of range of motion, thus leading to MO values closer to those found prior to treatment.

The present study did not set a cutoff point for trismus. We believed that assessment of the change in jaw mobility at different points in time would be more reliable, as it would enable identification of any restriction of range of jaw motion (whether inherent to the patient or resulting from the tumor or surgery) before the start of RT, thus ensuring that only RT-related changes would be analyzed. Many patients with UADT cancer exhibit limited MO prior to RT, whether as a result of surgery, due to extension of the tumor into the muscles of mastication, or due to reflex spasm of these muscles (17,28). This symptom tends to improve or resolve during RT, but it can gradually return as an adverse effect of RT (28).

This study corroborates the existing literature (17,23) in that patients with decreased functional ability exhibited a greater reduction in MO. We also found that, unlike in some studies (20,21), mandibular hypo mobility can develop regardless of radiation field and disease stage if the patient has reduced functional ability and develops mucositis during treatment. Therefore, although patients with hypo pharyngeal and laryngeal cancer have a lower incidence of mandibular hypo mobility (8), they must not be excluded from studies and prevention protocols, as they are likely to develop this complication during RT due to onset of mucositis and changes in diet.

Given the small number of patients who underwent IMRT and 3D-RT in our sample, no inferences could be drawn. Some studies (5,10) have suggested a lower incidence of mandibular hypo mobility with IMRT, but there is no consensus on this matter (21). We recommend that prospective studies with larger samples and longer follow-up be conducted to assess different modalities of RT and their impacts on jaw mobility. Protocols for prevention of mucositis and mandibular hypo mobility must be tested to ascertain whether they have a positive impact on jaw mobility and patient quality of life.

## Conclusion

The findings of this study suggest that patients with UADT cancer undergoing radiation therapy experience reduced jaw mobility. This complication occurs most frequently in patients with mucositis and reduced functional ability.

## References

[1] Parkin DM (2001). Global cancer statistics in the year 2000. Lancet Oncol.

[2] Warnakulasuriya S (2009). Global epidemiology of oral and oropharyngeal cancer. Oral Oncol.

[3] Mezitis M, Rallis G, Zachariades N (1989). The normal range of mouth opening. J Oral Maxillofac Surg.

[4] Khare N, Patil SB, Kale SM, Sumeet J, Sonali I, Sumeet B (2012). Normal mouth opening in an adult Indian population. J Maxillofac Oral Surg.

[5] Bensadoun RJ, Riesenbeck D, Lockhart PB, Elting LS, Spijkervet FK, Brennan MT (2010). A systematic review of trismus induced by cancer therapies in head and neck cancer patients. Support Care Cancer.

[6] Goldstein M, Maxymiw WG, Cummings BJ, Wood RE (1999). The effects of antitumor irradiation on mandibular opening and mobility: a prospective study of 58 patients. Oral Surg Oral Med Oral Pathol Oral Radiol Endod.

[7] Dijkstra PU, Kalk WW, Roodenburg JL (2004). Trismus in head and neck oncology: a systematic review. Oral Oncol.

[8] Weber C, Dommerich S, Pau HW, Kramp B (2010). Limited mouth opening after primary therapy of head and neck cancer. Oral Maxillofac Surg.

[9] Pauli N, Johnson J, Finizia C, Andrell P (2013). The incidence of trismus and long-term impact on health-related quality of life in patients with head and neck cancer. Acta Oncol.

[10] Chen YY, Zhao C, Wang J, Ma HL, Lai SZ, Liu Y (2011). Intensity-modulated radiation therapy reduces radiation-induced trismus in patients with nasopharyngeal carcinoma: a prospective study with >5 years of follow-up. Cancer.

[11] Schag CC, Heinrich RL, Ganz PA (1984). Karnofsky performance status revisited: reliability, validity, and guidelines. J Clin Oncol.

[12] Grandi G, Silva ML, Streit C, Wagner JC (2007). A mobilization regimen to prevent mandibular hypomobility in irradiated patients: an analysis and comparison of two techniques. Med Oral Patol Oral Cir Bucal.

[13] Richiardi L, Corbin M, Marron M, Ahrens W, Pohlabeln H, Lagiou P (2012). Occupation and risk of upper aerodigestive tract cancer: the ARCAGE study. Int J Cancer.

[14] Negri E, Boffetta P, Berthiller J, Castellsague X, Curado MP, Dal Maso L (2009). Family history of cancer: pooled analysis in the International Head and Neck Cancer Epidemiology Consortium. Int J Cancer.

[15] Dworkin SF, LeResche L (1992). Research diagnostic criteria for temporomandibular disorders: review, criteria, examinations and specifications, critique. J Craniomandib Disord.

[16] Peterson DE, Bensadoun RJ, Roila F (2011). Management of oral and gastrointestinal mucositis: ESMO Clinical Practice Guidelines. Ann Oncol.

[17] Johnson J, van As-Brooks CJ, Fagerberg-Mohlin B, Finizia C (2010). Trismus in head and neck cancer patients in Sweden: incidence and risk factors. Med Sci Monit.

[18] Wünsch-Filho V (2002). The epidemiology of oral and pharynx cancer in Brazil. Oral Oncol.

[19] Conway DI, Petticrew M, Marlborough H, Berthiller J, Hashibe M, Macpherson LM (2008). Socioeconomic inequalities and oral cancer risk: a systematic review and meta-analysis of case-control studies. Int J Cancer.

[20] Wetzels JW, Merkx MA, de Haan AF, Koole R, Speksnijder CM (2014). Maximum mouth opening and trismus in 143 patients treated for oral cancer: A 1-year prospective study. Head Neck.

[21] Louise Kent M, Brennan MT, Noll JL, Fox PC, Burri SH, Hunter JC (2008). Radiation-induced trismus in head and neck cancer patients. Support Care Cancer.

[22] Murphy BA, Beaumont JL, Isitt J, Garden AS, Gwede CK, Trotti AM (2009). Mucositis-related morbidity and resource utilization in head and neck cancer patients receiving radiation therapy with or without chemotherapy. J Pain Symptom Manage.

[23] Bragante KC, Nascimento DM, Motta NW (2012). Evaluation of acute radiation effects on mandibular movements of patients with head and neck cancer. Rev Bras Fisioter.

[24] Trotti A, Bellm LA, Epstein JB, Frame D, Fuchs HJ, Gwede CK (2003). Mucositis incidence, severity and associated outcomes in patients with head and neck cancer receiving radiotherapy with or without chemotherapy: a systematic literature review. Radiother Oncol.

[25] Shenoy VK, Shenoy KK, Rodrigues S, Shetty P (2007). Management of oral health in patients irradiated for head and neck cancer: a review. Kathmandu Univ Med J (KUMJ).

[26] Wang CJ, Huang EY, Hsu HC, Chen HC, Fang FM, Hsiung CY (2005). The degree and time-course assessment of radiation-induced trismus occurring after radiotherapy for nasopharyngeal cancer. Laryngoscope.

[27] Libshitz HI, DuBrow RA, Loyer EM, Charnsangavej C (1996). Radiation change in normal organs: An overview of body imaging. Eur Radiol.

[28] Ichimura K, Tanaka T (1993). Trismus in patients with malignant tumours in the head and neck. J Laryngol Otol.

